# Appropriate Anesthesia Regimen to Control Sevoflurane-Induced Emergence Agitation in Children; Propofol–Lidocaine and Thiopental Sodium–Lidocaine: A Randomized Controlled Trial

**DOI:** 10.5812/ircmj.16388

**Published:** 2014-07-05

**Authors:** Poupak Rahimzadeh, Seyed Hamid Reza Faiz, Mahmood Reza Alebouyeh, Azadeh Dasian, Azadeh Sayarifard

**Affiliations:** 1 Department of Anesthesiology and Pain Medicine, Rasoul-Akram Medical Center, Iran University of Medical Sciences, Tehran, IR Iran; 2 Preventive and Community Medicine specialist, Community Based Participatory Research Center, Iranian Institute for Reduction of High-Risk Behaviors, Tehran University of Medical Sciences, Tehran, IR Iran

**Keywords:** Lidocaine, Propofol, Sevoflurane, Thiopental Sodium, Emergence Agitation

## Abstract

**Background:**

Emergence Agitation (EA) is a common problem in pediatric anesthesia. The current study evaluated the effect of intravenous lidocaine combined with propofol or thiopental sodium to control EA by sevoflurane in children.

**Objectives:**

The current study aimed to compare the effectiveness of two anesthesia regimen propofol–lidocaine and thiopental sodium lidocaine to control sevoflurane-induced emergence agitation in children.

**Patients and Methods:**

The study enrolled 120 children aged 12 to 36 months with retinoblastoma who underwent induction of anesthesia with sevoflurane for Eye Examination Under Anesthesia (EUA). Sampling was done at Rasoul-Akram Hospital in Tehran, Iran. The subjects were randomly assigned into four groups including: group one (thiopental sodium-lidocaine [TL]), group two (thiopental sodium-saline [TS]), group three (propofol-lidocaine [PL]), and group four (propofol-saline [PS]). Emergence agitation was assessed by using a five-point scoring scale, every 10 minutes during the first 30 minutes after admission to the recovery room.

**Results:**

EA occurred in 24 cases (20%) of children. Incidence of EA in the TS, TL, PS, and PL groups were 21 (70%), 2 (6.7%), 1 (3.3%), and 0 (0%), respectively (P < 0.001). Nausea and vomiting after anesthesia did not occur in any of the patients. After removal of the endotracheal tube, laryngospasm complication occurrence in the TS group (10 cases) was higher than the other groups and no statistically significant difference was observed (P = 0.1).

**Conclusions:**

Propofol–lidocaine anesthesia regimen was more effective to control the pediatric emergence agitation than the other combinations.

## 1. Background

Retinoblastoma is one of the most important types of eye tumors. In addition to the treatment, this tumor requires periodic progress assessment and thus children undergo frequent general anesthesia for eye examination and sevoflurane is mostly used for this purpose (1). Sevoflurane leads to Emergence Agitation (EA) complication in children (2, 3). Increased use of sevoflurane in developing countries has increased EA incidence rate (4). Eckenhaff described EA for the first time in early 1960s (5).

Major characteristics of EA include delirium, hallucinations, excitation, and anxiety which occur in children with different manifestations such as crying and self-injury and involuntary physical activities occur early after surgery (6, 7). Incidence of sevoflurane-induced EA varies from 20% to 80%; it is often observed in preschoolers and depends on the used anesthesia and scoring system (8, 9). Risk factors of EA incidence are related to anesthesia, surgery, and the patient susceptibility. Sevoflurane-induced agitation affects the central nervous system and induces seizures and behavioral changes after the surgery. Eye and autorhinolarynx related surgeries, younger age, pre-operative anxiety, no background of surgery, and adjustment disorders in patients are risk factors for the incidence of EA (10).

Despite spontaneous recovery, emergence agitation is yet considered as a potentially serious complication due to the risks of self-injury and stress both for the family and caregivers. Thus, the effect of various medications such as midazolam, propofol, ketamine and alpha-2 agonists was investigated to reduce the pediatric agitation (11-13). Lidocaine is one of the medications, which its effectiveness via intravenous or local use is raised in the recent studies. Preoperative lidocaine could achieve preemptive analgesia and reduction of airway reflexes for pediatric patients undergoing general anesthesia with sevoflurane and cause less EA without excessive sedation in the recovery room (14, 15).

## 2. Objectives

The current study aimed to compare the effectiveness of two anesthesia regimens propofol–lidocaine and thiopental sodium–lidocaine to control sevoflurane-induced emergence agitation in children.

## 3. Patients and Methods

The current study was conducted as a double blind randomized clinical trial confirmed by the Ethics Committee of Tehran University of Medical Sciences and was registered in IRCT center (IRCT2013072914199N1). Children aged 12 to 36 months with eye retinoblastoma formed the study population. Inclusion criteria include children with ASA (American Society of Anesthesiologists) class one and two and their willingness to participate in the study. Participants’ parents signed the written consent as well. Patients with seizures background, any type of liver, kidney, lung and heart diseases, catching colds during the past four weeks, allergy to lidocaine, and children with severe crying were excluded from the study.

An incidence of postoperative agitation of 40% or more after sevoflurane anesthesia (16) and a reduction in EA up to 10% (15) were considered and then it was calculated that 30 patients were required in each group (for the 0.05 level of significance and a power of 0.80). The sampling was convenient. The enrolled patients were selected from the patients who were referred to Rasoul-Akram Hospital, a referral university general hospital in Tehran, Iran, based on meeting all inclusion and exclusion criteria and their written informed consent. The samples were completed in a period of 11 months (Oct. 2012 to Sep. 2013).

To conduct the double-blind study, the patients and the persons who evaluate the final outcome (agitation) should not to be aware of the prescribed medication. Patients were assigned to one of the four groups by a computer-derived randomization list as follows:

one: thiopental sodium–lidocaine (TL),
two: thiopental sodium–saline (TS),
three: poropofol–lidocaine (PL) and
four: propofol–saline (PS).

A questionnaire including demographic information and the study variables such as age, sex, weight, duration of anesthesia, and duration of examination under anesthesia was used to collect the data. Children received no pre-medications and underwent standard monitoring including ECG, blood pressure, end-tidal CO2 and pulse oximetry measurements during the surgery with calibrated Saadat devices (Masimo, Irvine, CA). General anesthesia induction was performed with inhalation method using sevoflurane 5%. After appropriate venipuncture for children, group one received 5 mg/kg thiopental sodium and 1 mg/kg lidocaine injection, group two received 5 mg/kg thiopental sodium injection mixed with saline, group three received 2 mg/kg propofol and 1 mg/kg lidocaine injection, and group four received 2 mg/kg propofol injection mixed with saline. Then, appropriate Laryngeal Mask Airway (LMA) in terms of size was situated, and anesthesia was maintained by sevoflurane 3% and 5 L/min oxygen. Lung ventilation was manually controlled and end-tidal CO2 was maintained between 30 and 35 mmHg. After the operation sevoflurane discontinued and LMA was removed when the patient resumed adequate spontaneous breathing. The patient was transferred to the recovery unit and the incidence of EA was evaluated by the anesthesiologist without knowledge of the child`s group. EA was assessed according to Cole grading scale:

1. asleep,
2. awake and calm,
3. irritable or consolable crying,
4. inconsolable crying, and
5. severe restlessness.

Statistically, children with scores of four or five were classified as agitated (15). The evaluation was done every 10 minutes during the first 30 minutes after admission to the recovery room. Complications such as nausea and vomiting and laryngospasm were also recorded.

Collected data were analyzed by SPSS Software and STATA. Continuous data were presented as mean ± SD. Data analysis was performed using Chi-square, Fisher Exact tests, T-test and ANOVA. To compare the agitation scores in the four groups at different times after anesthesia, repeated measure ANOVA was used. To assess emergence agitation scores of four (inconsolable crying) or five (severe restlessness) were considered as incidence of emergence agitation. Therefore, dependent variable (agitation score) was converted to a dichotomous variable with agitation, and without agitation, and then to compare EA in the four groups Chi-square or Fisher Exact tests were used. P-Value less than 0.05 was considered statistically significant.

## 4. Results

**Figure 1.**
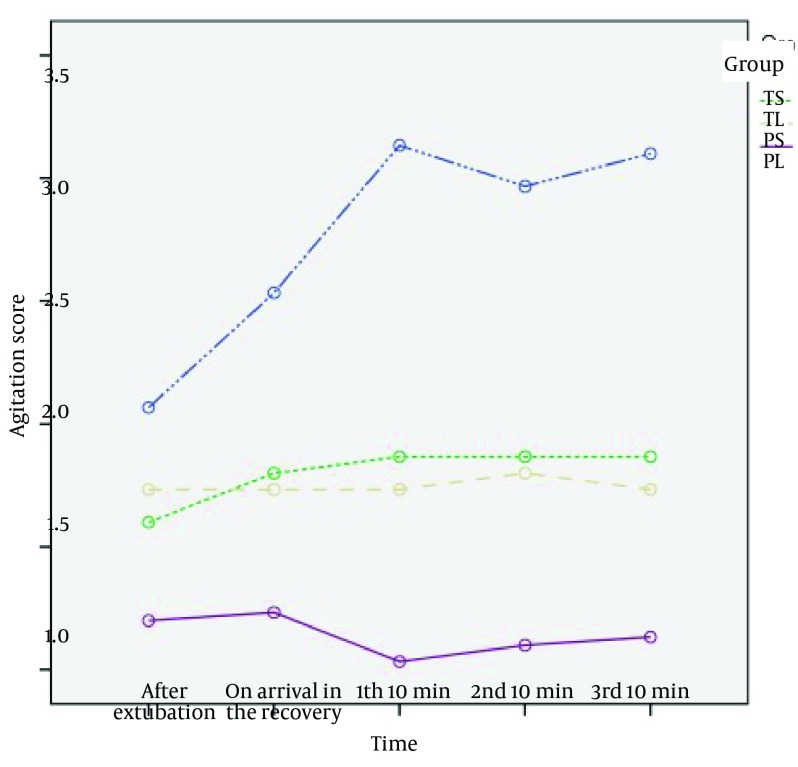
Comparison of Agitation Scores in the Four Groups

Overall 128 children were included in the study. Final analysis was done on 120 children. Eight cases, four from group TL, two from group TS, two from group PS, were excluded due to incomplete data. Characteristics of the patients including age, sex, weight, duration of anesthesia, and duration of examination under anesthesia are given in Table 1. There was no significant difference between the groups, which suggests appropriate randomization of patients in the four groups of the study. Agitation score in PL group was the lowest and then PS and TL groups followed, and the highest score was in TS group (P < 0.001) (Figure 1).

**Table 1.** Patient Demographic and Clinical Data ^a,b^

	TS Group (n = 30)	TL Group (n = 30)	PS Group (n = 30)	PL Group (n = 30)	P Value
**Age, mo**	29 ± 9.2	30 ± 7.2	31.6 ± 6.4	26.9 ± 8.2	0.2
**Sex, male/female**	8/22	14/16	16/14	16/14	0.12
**Weight, kg**	14.7 ± 4	15.2 ± 3.2	13.7 ± 2.3	13.3 ± 3.1	0.12
**Duration of anesthesia, min**	9.2 ± 22.2	6.4 ± 20.6	11.5 ± 27.2	9.3 ± 23	0.11
**Duration of examination, min**	9.2 ± 17.5	15.2 ± 6.1	6.5 ± 16.3	4.1 ± 12.8	0.11

^a^ Data are expressed as mean ± SD or number of patients.

^b^ Abbreviations: TS, thiopental anesthesia with isotonic salin injection; TL, thiopental anesthesia with lidocaine injection; PS, propofol anesthesia with isotonic salin injection; PL, propofol anesthesia with lidocaine injection.

Table 2 shows Post HOC test (Bonferroni) for pairwise comparisons of agitation intensity in the four groups. To assess emergence agitation score four (Inconsolable crying) or five (severe restlessness) were considered as the incidence of emergence agitation. Out of the 120 subjects, 24 cases (20%) had EA. The incidence of EA in the TS, TL, PS, and PL groups were 21 (70%), 2 (6.7%), 1 (3.3%), and 0 (0%), respectively. There was statistically significant difference regarding EA incidence in the four groups (P < 0.001).

**Table 2.** Post HOC Test for Pairwise Comparisons of Agitation Intensity in the four Groups ^a^

(I) Group	(J) Group	Mean Difference (I-J)	Sig.	95% Confidence Interval	
				Lower Bound	Upper Bound
**TS**					
	TL	0.96^b^	0.000	0.54	1.38
	PS	1.01^b^	0.000	0.59	1.43
	PL	1.62^b^	0.000	1.20	2.04
**TL**					
	TS	-0.96^b^	0.000	-1.38	-0.54
	PS	0.05	1.000	-0.37	0.47
	PL	0.66^b^	0.000	0.24	1.08
**PS**					
	TS	-1.01^b^	0.000	-1.43	-0.59
	TL	-0.05	1.000	-0.47	0.37
	PL	0.61^b^	0.001	0.19	1.03
**PL**					
	TS	-1.62^b^	0.000	-2.04	-1.20
	TL	-0.66^b^	0.000	-1.08	-0.24
	PS	-0.61^b^	0.001	-1.03	-0.19

^a^ Abbreviations: TS, thiopental anesthesia with isotonic salin injection; TL, thiopental anesthesia with lidocaine injection; PS, propofol anesthesia with isotonic salin injection; PL, propofol anesthesia with lidocaine injection.

^b^ The mean difference is significant at 0.05 level.

Agitation incidence in the four groups was recorded at different times after anesthesia, and as in Table 3, agitation in the TS group was significantly higher than other groups at all times (P < 0.001). Incidence of agitation in the four groups is given in Table 4 in terms of patient gender, in which no significant difference was observed.

**Table 3.** Occurrence of Emergence Agitation With the Lapse of Time in the Patients ^a,b^

	TS Group (n = 30)	TL Group (n = 30)	PS Group (n = 30)	PL Group (n = 30)	P Value
**After extubation**	9 (30)	2 (6.2)	0 (0)	0 (0)	< 0.001
**On arrival in the recovery room**	13 (43.3)	0 (0)	0 (0)	0 (0)	< 0.001
**1st 10 min **	19 (63.3)	0 (0)	0 (0)	0 (0)	< 0.001
**2nd 10 min **	10 (33.3)	0 (0)	1 (3.3)	0 (0)	< 0.001
**3rd 10 min **	10 (33.3)	0 (0)	0 (0)	0 (0)	< 0.001

^a^ Values are number (%) of patients that developed emergence agitation.

^b^ Abbreviations: TS, thiopental anesthesia with isotonic saline injection; TL, thiopental anesthesia with lidocaine injection; PS, propofol anesthesia with isotonic saline injection; PL, propofol anesthesia with lidocaine injection.

**Table 4.** Incidence of Emergence Agitation by Sex ^a^

	TS Group (n = 30)	TL Group (n = 30)	PS Group (n = 30)	PL Group (n = 30)
**Female**	5 (23.8)	1 (50)	0 (0)	1 (100)
**Male**	16 (76.2)	1 (50)	0 (0)	0 (0)
**P value**	0.58	0.92	-	0.34
**Total**	21	2	0	1

^a^ data are presented as No. (%).

Average weight in children with EA was lower than those of the children without EA. (14.3 ± 3.3 vs. 13.9 ± 4.2 kg), which was not statistically significant (P = 0.73). Average age of the children with EA was lower than that of the children without EA (27 ± 9.4 vs. 29.9 ± 7.4 years), which was not statistically significant (P = 0.18).

Nausea and vomiting after anesthesia did not occur in any of the patients. After removing the endotracheal tube, laryngospasm complication occurred in 10 cases (33.3%) in the TS group, six cases (20%) in the PS group, four cases (13.3%) in the TL group, and 3 cases (10%) in the PL group, and no significant statistical difference was observed (P = 0.1).

## 5. Discussion

The current study aimed to investigate the effect of intravenous lidocaine combined with propofol or thiopental sodium in controlling anesthesia agitation by sevoflurane in children with retinoblastoma. EA incidence by sevoflurane has been confirmed in many studies (5, 6). There are various scoring tools to evaluate EA (17-19), however, there is no consensus over a single scoring system. Cole scoring system was used in the current study (18) which seemed to be an appropriate and practical scale for EA evaluation in children, especially in the early postoperative period (14, 15).

In the current study, EA incidence was observed in 20% of the children. Voepel-Lewis et al. (9) reported the incidence of EA as 18% in three to seven year old children who underwent general anesthesia for different procedures. Saringcarinkul et al. (16) reported EA as 43.2% in children who underwent general anesthesia for different surgeries. In a study in South Korean, EA incidence was reported as 18.8% in children who underwent strabismus surgery (15).

Mizuno et al. (10) introduced EA incidence risk factors as sevoflurane anesthesia, eye-related surgeries and low age. Although factors for the incidence of sevoflurane-induced EA have not been certainly recognized, rapid elimination of sevoflurane due to its low solubility in blood was mentioned as EA reason in some patients (20). The other effective factors in etiology of EA incidence in children are difficult parental-separation behavior (16), child's inability to adapt to sudden given changes ,an unfamiliar environment after awakening, immature neurologic development, and increased pain feeling (2, 21).

Clinically, in preschool-aged children, often EA related behavior cannot be distinguished from pain related behavior (15). Pain is considered as a major factor in relation with EA incidence (14, 15). Sheard et al. (22) showed that sub-Tenon lidocaine injection significantly reduces pain after surgery, oculocardiac reflex and nausea and vomiting in children undergoing strabismus surgery. Sub-Tenon injection is an acceptable anesthetic technique in eye surgery in adults (23). However, since it requires cooperation by the patient it is not often used for children (15). Therefore, the current research studied the effect of intravenous injection of lidocaine in children. The present study showed that the lowest incidence of EA was in the propofol-lidocaine group.

Seo et al. (15) and Elgebaly (14) showed that sub-Tenon lidocaine injection is effective to reduce the incidence of EA in children undergoing strabismus surgery. Hwang et al. (24) compared the incidence of sevoflurane-induced EA in 4 to10 year old candidates for elective Tonsillectomy surgery in two groups who received propofol or thiopental sodium. Similar to the current study, their findings showed that propofol was more effective in reducing the incidence of EA compared to thiopental sodium. Kim et al. (25) indicated that low dosage of propofol or fentanyl can reduce the incidence of sevoflurane-induced agitation. Aouad et al. (26) showed the effectiveness of propofol in reduced incidence of sevoflurane-induced EA in children undergoing strabismus surgery. Also, parents of the group receiving propofol were more satisfied.

Propofol-induced recovery time was longer than that of sevoflurane, thus its resulting anesthesia caused less EA incidence compared to that of sevoflurane (13). Reduced incidence of EA can be due to residual sedative effect and euphoric effect of propofol in the early recovery period as well (27). EA has a peak incidence in the first 30 minutes after anesthesia (17). In the current study, EA was higher in the group receiving thiopental todium–saline in all recorded times (P < 0.001). In lidocaine receiving groups (PL, TL) only two children were caught by EA after extubation. In Seo's study, EA incidence was observed in the first 10 minutes after recovery in most patients (72%) and in the group which received lidocaine. No cases of EA were observed at the first 10 minutes of recovery (15).

The current study also showed that the effect of lidocaine and propofol on the reduced incidence of EA in children had no relation with gender, weight and age of the child. In the current study, nausea and vomiting did not occur in the patients. Hwang et al. (24) and Coolong et al. (28) indicated that propofol and thiopental sodium do not make significant difference in the incidence of nausea and vomiting in patients. On the other hand, Kim et al. (25) showed that propofol causes lower nausea and vomiting in patients compared to fentanyl.

Incidence of laryngospasm was higher in the group receiving thiopental sodium- saline compared to the other groups, in which the difference was not significant. Laryngospasm is more common in pediatric patients with inadequate depth of anesthesia during emergence. More laryngospasm was observed with sodium thiopental. Erb et al. (29) demonstrated that fentanyl was not able to reduce laryngospasm incidence in the children who were anesthetized with sevoflurane.

One strong point in the current study was appropriate randomization of children in the four groups. One of the limitation was that children were examined periodically which was a limitation for the work, and many children had to be excluded from the study in order to prevent repetition of the patients. Also because of lower age it was difficult to have verbal contact; therefore, the evaluation was hard to detect.

Overall, findings in the current study indicated that however it was useful to add lidocaine to induction drugs on emergence agitation reduction but propofol-lidocaine anesthesia regimen reduced the incidence of sevoflurane-induced agitation in children with retinoblastoma more than the other groups. It is recommended to perform more studies to investigate the effects of lidocaine with different dosages and used methods with larger sample size to study EA incidence in children who are anesthetized with sevoflurane.
